# Supplementary material for: Reactive centre loop dynamics and serpin specificity
Source: Sci Rep. 2019 Mar 7;9:3870. doi: 10.1038/s41598-019-40432-w (PMC6405850; doi:10.1038/s41598-019-40432-w)
Supplement: Supplementary file 1 — Supporting Information [file 41598_2019_40432_MOESM1_ESM.pdf]

Supporting Information

Reactive Centre Loop Dynamics and Serpin Specificity

Emilia M. Marijanovic, James Fodor, Blake T. Riley, Benjamin T. Porebski, Mauricio G. S. Costa, Itamar Kass, David E. Hoke, Sheena McGowan, Ashley M. Buckle

SI Tables

Table S1: *Data collection and refinement statistics.*

Data collection	Native conserpin-AAT_{RCL}
Wavelength (Å)	0.9537
Space group	C 2 2 2_1
Unit cell dimensions (Å)	68.06, 75.28, 150.45, 90, 90, 90
Resolution (Å)	2.48
Number of measured reflections	13376
Number of unique reflections	13455
Completeness (%)	95.47
Redundancy	29.2
R_{pim}	
<I/σI>	99.73
Structure refinement	
Number of reflections	13376
Number of protein atoms	2561
Number of water molecules	9
R_{work} (%)	0.1986
R_{free} (5% of data) (%)	0.2593

CC1/2	0.998
CC*	1
RMSD bond lengths (Å)	0.008
RMSD bond angles (°)	1.17
**Average B-factor** (Å^2)	82.60
Protein	82.70
Solvent	59.30
Ramachandran	
Favoured (%)	91
Outliers (%)	0.57
MolProbity score	2.32, 85th percentile* (N=6912, 2.48Å ± 0.25Å)
PDB ID	6EE5

SI Figures

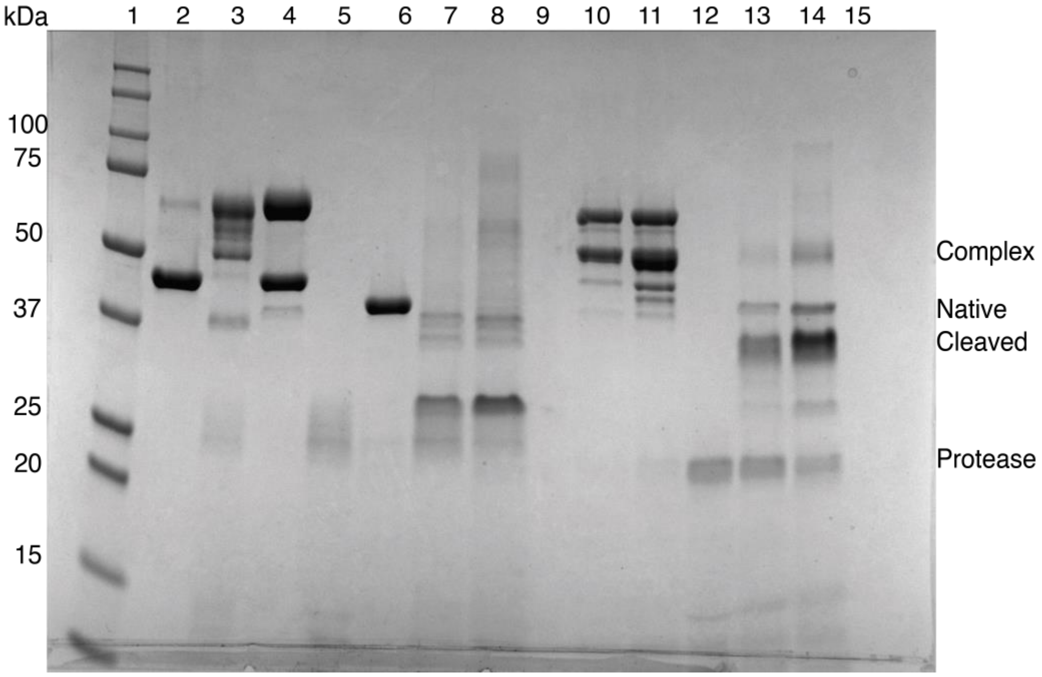

Fig. S1. Full length (uncropped) SDS-PAGE gel shown in Fig. 1. The gel in Fig. 1. was cropped from lane 1-8. Lanes 9-14 show the formation of a serpin: protease complex between α1-AT and conserpin-AAT$_{RCL}$ with trypsin. From left to right: 1. Molecular weight markers (kDa); 2. α1-AT alone; 3. 1:1 ratio of α1-AT: HNE; 4. 2:1 ratio of α1-AT:HNE; 5. HNE alone; 6. conserpin-AAT$_{RCL}$ alone; 7. 1:1 ratio of conserpin-AAT$_{RCL}$:HNE; 8. 2:1 ratio of conserpin-AAT$_{RCL}$:HNE; 9. blank; 10. 1:1 ratio of α1-AT: trypsin; 11. 2:1 ratio of α1-AT: trypsin; 12. Trypsin alone; 13. 1:1 ratio of conserpin-AAT$_{RCL}$:trypsin; 14. 2:1 ratio of conserpin-AAT$_{RCL}$:trypsin; 15. blank.

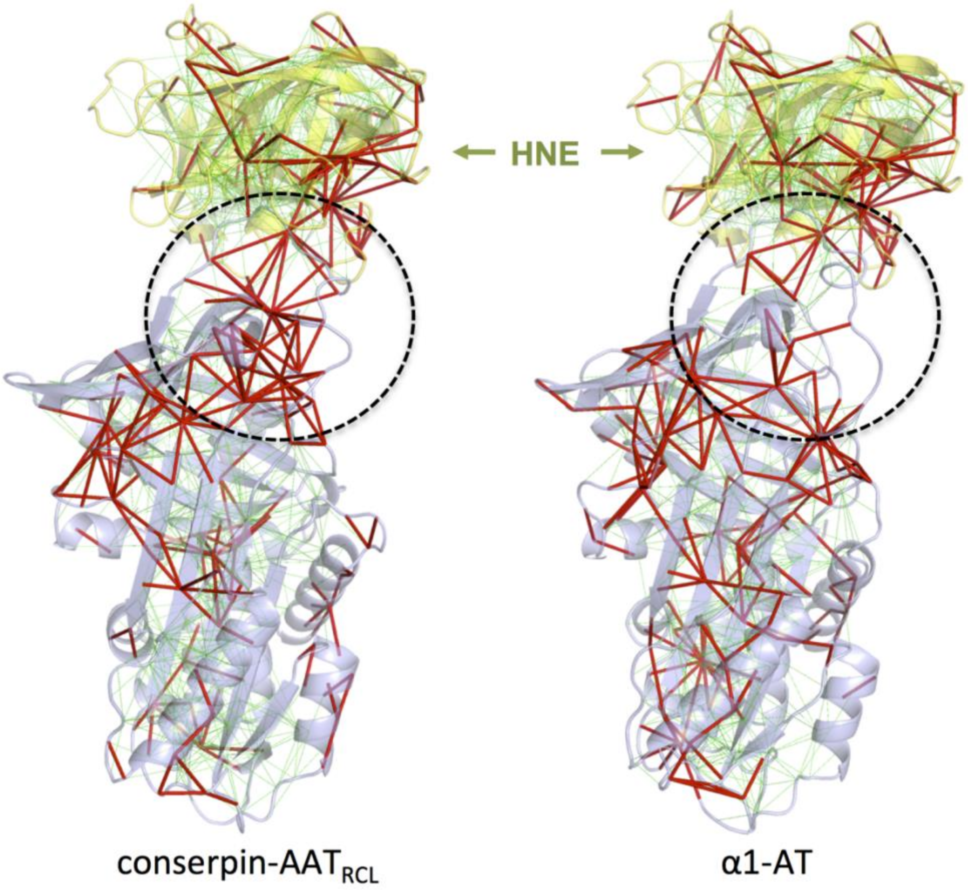

Fig. S2. Visualization of the frustration networks mapped onto the modeled complexes between conserpin-AAT$_{RCL}$ or α1-AT with HNE (left and right, respectively). Minimally and highly frustrated contacts are represented in green and red, respectively.

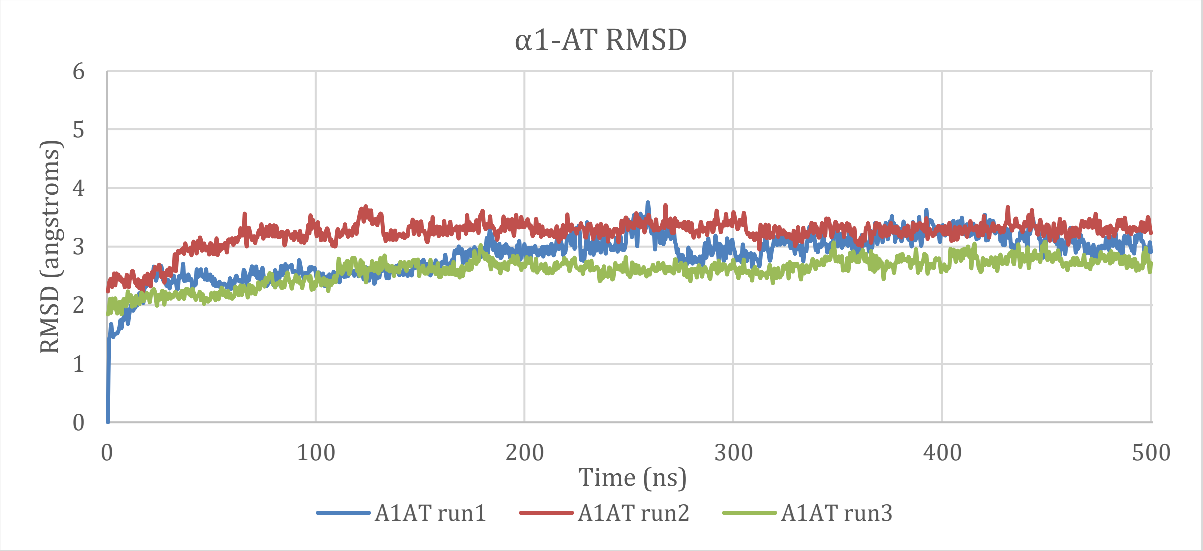

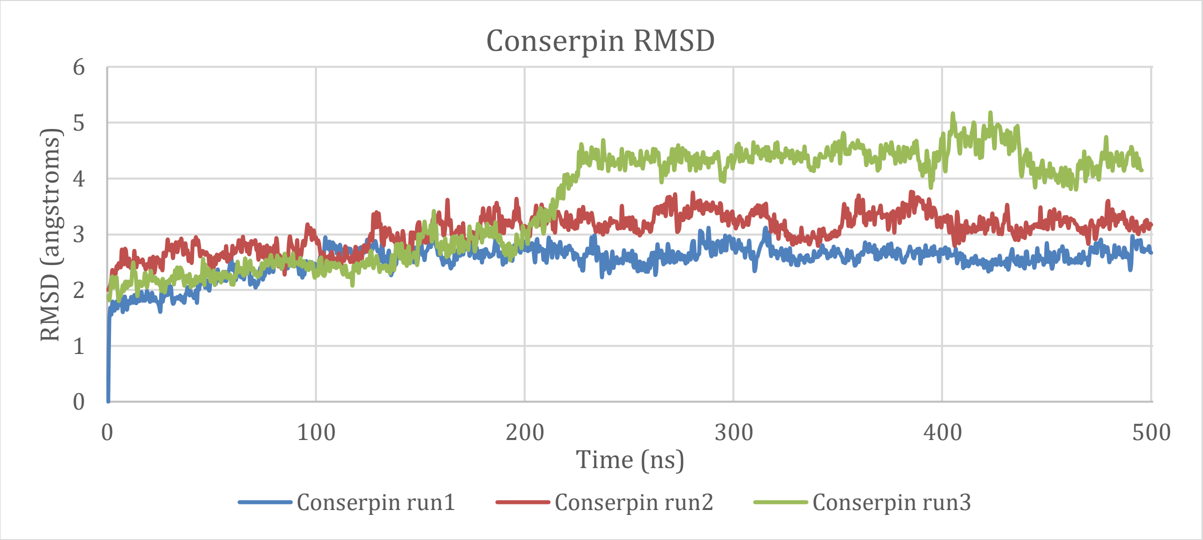

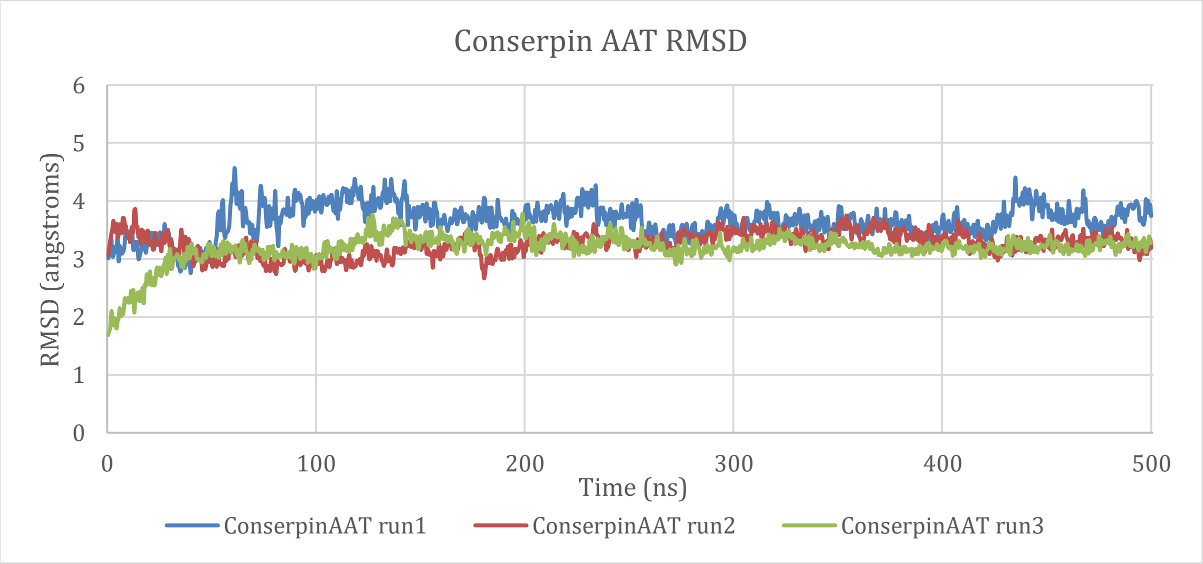

Fig. S3. Root Mean Squared Deviation (RMSD) plots for all systems. The RMSD for for α1-AT (top), conserpin (middle), and conserpin-AAT (bottom), showing all three runs

separately. All systems converge to around 3-4 angstroms after about 150 ns, with the exception of conserpin run 3, which converges to an RMSD of about 4.5 angstroms after 220 ns.

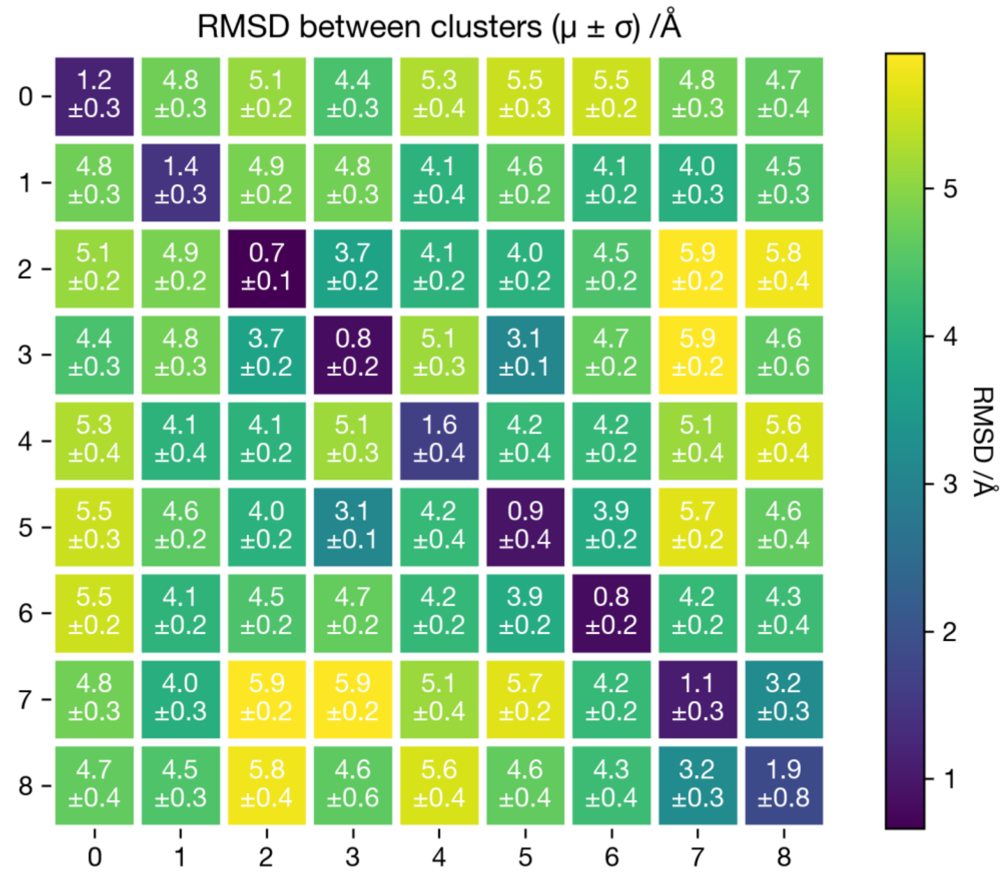

Fig. S4. Average cross-cluster RMSD heatmap. After clustering, a set of exemplar structures was extracted from each cluster. Pairwise RMSDs were calculated for all frames within a cluster, and also all frames between clusters. These pairwise RMSDs were then averaged, and displayed here as a heatmap. The low RMSD along the main diagonal shows that the exemplar structures from each cluster are self-similar, and the large RMSD outside the main diagonal shows that each cluster is distinct to all other clusters.

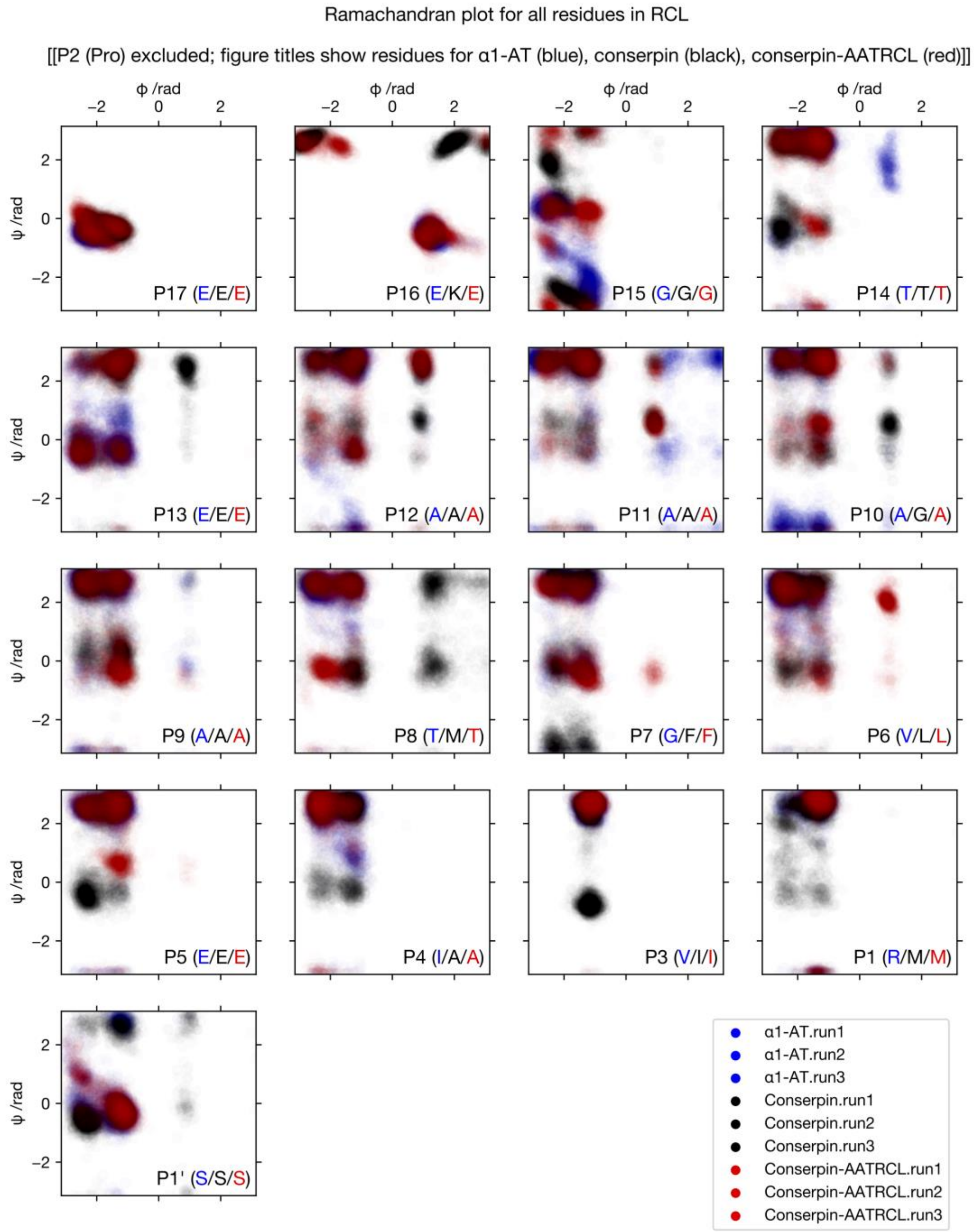

Fig. S5. Ramachandran plots show changes in dynamics of the RCL (P17-P1'). to localize conformational differences between each system on a residue-by-residue basis, phi and psi angles were calculated for all residues (excluding P2 Proline), and plotted here. The conformations explored in α1-AT simulations are coloured blue; in conserpin simulations, black; in conserpin-AAT$_{RCL}$ simulations red. In particular, it shows that α1-AT and conserpin-AAT$_{RCL}$ occupy a Lα torsion at P16 (bent hinge), whereas conserpin does not. contributing to

the insertion of the hinge region. Additionally, conserpin is prone to forming α-helices between P5−P3, in contrast to the other two groups.
